# Tissue-Specific Hormonal Variations in Grapes of Irrigated and Non-irrigated Grapevines (*Vitis vinifera* cv. “Merlot”) Growing Under Mediterranean Field Conditions

**DOI:** 10.3389/fpls.2021.621587

**Published:** 2021-02-01

**Authors:** Camila Ribalta-Pizarro, Paula Muñoz, Sergi Munné-Bosch

**Affiliations:** ^1^Department of Evolutionary Biology, Ecology and Environmental Sciences, University of Barcelona, Barcelona, Spain; ^2^Research Institute of Nutrition and Food Safety, University of Barcelona, Barcelona, Spain

**Keywords:** grape berries, cytokinin, non-climacteric fruit ripening, fruit growth and development, water deficit

## Abstract

Agricultural practices in grapevines management include water restrictions due to its positive effect on wine quality, especially when applied at fruit ripening. Although the effects of water stress in some groups of phytohormones have already been described in leaves and whole grapes, information regarding tissue-specific variations in hormones during ripening in grapes is scarce. Field-grown grapevines from the cv. “Merlot” were subjected to two differential water supplies, including only rainfed, non-irrigated vines (T0) and vines additionally irrigated with 25Lweek^−1^ vine^−1^ (T1). Tissue-specific variations in the hormonal profiling of grapes [including changes in the contents of abscisic acid (ABA), jasmonic acid (JA), salicylic acid (SA), the ethylene precursor 1-amino-cyclopropane-1-carboxylic acid (ACC), the auxin indole-3-acetic acid, gibberellins 1, 3, 4, and 7 (GA_1_, GA_3_, GA_4_, and GA_7_), the cytokinins *trans*-zeatin, and 2-isopentenyl adenine, including as well their respective ribosylated forms] were periodically evaluated from *veraison* to harvest. The hormonal profiling in leaves was also measured at the beginning and end of the season for comparison. Results showed that grape growth dynamics were transiently affected by the differences in water regimes, the increased water supply leading to an accelerated growth, slightly reduced accumulation of sugars, and transiently lowered pH, although grape quality did not differ between treatments at harvest. Hormonal profiling of whole berries did not reveal any difference in the endogenous contents of phytohormones between treatments, except for a transient decrease in GA_4_ contents in T1 compared to T0 vines, which was not confirmed at the tissular level. Hormonal profiling at the tissue level highlighted a differential accumulation of phytohormones during ripening in berry tissues, with pulps being particularly poor in ABA, JA, and SA contents, seeds particularly accumulating ACC, gibberellins, and zeatin-type cytokinins, and the skin being particularly rich in auxin and active cytokinins. Changes in water supply led to very small and transient changes in the endogenous contents of phytohormones in the seeds, pulp, and skin of berries, the most remarkable variations being observed in cytokinin contents, which increased earlier [between 5 and 12days after *veraison* (DAV)] but later kept more constant in the skin from T1 compared to T0 vines and were also 3-fold higher at 40 DAV in seeds of T1 compared to T0 vines. It is concluded that small changes in water supply can trigger hormonal-driven physiological adjustments at the tissular level affecting the evolution of fruit growth and quality throughout grape berry ripening.

## Introduction

Wine grape (*Vitis vinifera* L.) is one of the most important fruit crops worldwide for both economic and cultural reasons, having Spain the largest cultivated area, positioned itself as the third in product turnover, and the main exporter of wines, based on volume, around the world (OIV, 2019). Obtaining high-quality grapes to elaborate high-quality wines has been a priority for the viticultural industry over decades and, therefore, many agricultural and winery practices have been devoted to it. Along with “Cabernet Sauvignon,” “Cabernet Franc,” “Malbec,” and “Petit Verdot,” “Merlot” is one of the primary grapes used in Bordeaux wine, it is one of the most popular red wine varietals in many markets and, consequently, it is one of the world’s most planted grape varieties (OIV, 2019). Agricultural practices in grapevines include various types of water management, such as regulated water-deficit, which is considered a key factor in many viticultural regions, especially in warm areas when applied at fruit ripening after *veraison* (Chaves et al., 2010; Cáceres-Mella et al., 2018). Water deficit during this period has shown to have positive effects on the quality of red wine grape varieties, since it improves the microclimate of fruit zone, reduces the size of berries, and increases the skin to pulp ratio, among other physiological responses that enhance phenolic compounds concentration, which indeed improves wine color, astringency, mouthfeel, bitterness, stability, and antioxidant activity (Santos et al., 2005; Castellarin et al., 2007; Deluc et al., 2009; Chaves et al., 2010). Water management is a key factor during grapevines life cycle, since it regulates carbon partitioning, vegetative growth, and the biochemical characteristics of berries (Deloire et al., 2004). The positive effect of water management in grapes production has been mostly attributed to endogenous increases of abscisic acid (ABA), which is considered as an important signal to trigger the onset of ripening in grape berries and has proved to have a key role in the modulation of secondary metabolism, for example, by the induction of gene expression of enzymes related to the phenylpropanoid pathway (Berli et al., 2015; Yukari et al., 2015; Villalobos-González et al., 2016; Ramírez et al., 2018).

Grape berries are non-climacteric fruits as they do not exhibit a large rise in ethylene production or respiration rate at the onset of ripening (*veraison*). However, ethylene may still play a role in berry development and ripening, as it has been shown with (2-chloroethyl)phosphonic acid, an ethylene-releasing reagent that delays ripening when applied early in berry development (Böttcher et al., 2013). Although the effects of water stress in the regulation of endogenous contents of ABA in grapevines has been widely studied, information regarding variations of other hormonal groups during grape berry ripening is still very poorly understood. In fact, fruit ripening is an intricate phenomenon in plant development where different groups of phytohormones have a specific function in time and space, resulting in a tight control of the process that affects sensory and nutritional characteristics, as well as the productivity and quality of fruits (Giovannoni, 2004). Currently, different studies have shown that grape berry ripening involves a complex hormonal control with ABA, ethylene, and brassinosteroids proposed as ripening promoters, while auxins, cytokinins, gibberellins, jasmonates, and polyamines as ripening inhibitors (Davies and Böttcher, 2009; Deluc et al., 2009; Kuhn et al., 2013; Fortes et al., 2015). Nevertheless, these studies have been focused on development of grape berries in vineyards with no variations in water regimes, and nowadays, in the current frame of global change, it is very important to know to what extend and how a differential water regime can influence hormonal variations and quality of grape berries. Furthermore, to our knowledge, there is no information regarding studies examining a putative differential hormonal contribution at the tissue level on the ripening process of grape berries.

Here, we aimed to examine endogenous variations of the hormonal profile in grape berries, with a focus on tissue-specific changes in hormonal contents during fruit ripening under different water supplies. We examined tissue-specific hormonal changes (in the skin, pulp and seeds) to establish putative correlative evidence of the hormonal regulation of the differential growth dynamics of grape berries caused by applying contrasting water regimes. Furthermore, the hormonal profiling of leaves was measured at the beginning and at the end of the study in order to compare the hormonal contents of skin, pulp, and seeds with that of leaves.

## Materials and Methods

### Plant Material, Treatments, and Sampling

The assays were established on 20-year-old “Merlot” grapevines (*Vitis vinifera* L.) on SO4 rootstock, in the organic commercial vineyard ArtCava winery located in Avinyonet del Penedés, Barcelona, Spain (41°22'06.3''N 1°46'22.9''E). Vines grew under Mediterranean climate conditions following a head-trained, spur-pruned training system with vines nearly north-south oriented, planted at 2m between rows and with 1m between them with a vineyard plant density of 5,000 vines per hectare. Vines were subjected to two different water regimes from the onset of *veraison* until harvest, one of them only with rainfall supply (T0) and the other one was manually irrigated with an additional water supply of 25Lweek^−1^ vine^−1^ (T1), corresponding to the 50% of daily crop evapotranspiration estimated for the area. The *veraison* date [August 10th, 2019 (Julian day 231)] was determined by visual observation of the vineyard. The experimental design consisted of completely randomized blocks with 10 replicates, where each replicate consisted of four consecutive vines. Each block corresponded to a different row and each treatment was distanced at least five vines within the same block. Treatments and replicates were randomly distributed among the experimental sites.

Berries samples for full berry and tissular analysis were collected at −2, 5, 12, 27, 40, and 54days after *veraison* (DAV), the latter as determined by the producer considering the visual change of color of the grape berries, more specifically when 50% of berries changed color (BBCH 85 of Lorenz scale, Lorenz et al., 1995). Last sampling data corresponded to the commercial harvest date, which occurred on October 3rd, 2019 (Julian day 276). All samplings were performed on clear sunny days at 1 pm. local time. At every sampling day, 100 berries per replicate were randomly collected, immediately frozen with liquid nitrogen, and stored at −80°C for further analysis. Likewise, four mature, non-senescing leaves were collected for analysis, two of them were immediately used for stress markers measurement and the other two immediately frozen in liquid nitrogen and stored at −80°C until hormonal analyses.

### Leaf Stress Parameters

For leaf water status estimation, leaves were weighed, and leaf area was measured using a flatbed scanner and an image processor (ImageJ 1.49 v, National Institutes of Health, Bethesda, MD, United States). Then, leaves were re-hydrated, stored at 4°C for 24h in darkness and weighed, for being subsequently oven-dried at 50°C until constant weight. Relative leaf water content (RWC) was determined as 100×(FW−DW)/(TW−DW), where FW is the fresh weight, TW is the turgid weight (after being hydrated), and DW is the dry weight, after being oven-dried. Leaf hydration (H) was calculated as (FW−DW)/DW and leaf mass area (LMA) ratio as DW/leaf area.

Proline content was determined as previously described by Bates et al. (1973) with some modifications. Briefly, 100mg of leaf tissue was weighted and grinded with liquid nitrogen to later extract with 750ml of 3% aqueous sulfosalicylic acid followed by vortex and centrifugation at 9,000rpm for 10min at 4°C. Supernatant was collected and the pellet re-extracted with 750ml of 3% aqueous sulfosalicylic acid. After vortexing and centrifugation, supernatants were pooled, and 100ml was reacted with 2ml of glacial acetic acid and 2ml of ninhydrin (prepared by warming 1.25g ninhydrin in 30ml glacial acetic acid and 20ml 6M phosphoric acid). Test tubes with the reaction mixture were incubated for 1h at 100°C and the reaction terminated at 4°C for 10min. Therefore, 1ml toluene was added to the mixture and after vigorous agitation, test tubes were set at room temperature for phase separation. The upper phase containing the chromophore was recovered and the absorbance read at 520nm using toluene for a blank. A standard curve of proline was performed following the same protocol to determine sample concentration.

The maximum efficiency of photosystem II photochemistry (*F*v/*F*m ratio), which in an indicator of photoinhibitory damage to the photosynthetic apparatus, was estimated in leaves that were exposed to darkness for at least 1h by using a portable fluorimeter Mini-PAM (Walz, Effeltrich, Germany), as described by van Kooten and Snel (1990).

### Grape Berry Quality

In grapes, the following physicochemical variables were assessed: Weight and volume of 50 berries, titratable acidity (TA, g tartaric acid L^−1^), pH, total soluble solids (TSS, °Brix), and TSS/TA in berry juice following the standardized methodology described by the International Organization of vine and Wine (OIV, 2012).

### Hormonal Profiling

Leaf, whole berry, and berry tissues (including the skin, the pulp, and the seeds, which were manually separated with the help of a scalpel from the fruit while using liquid nitrogen to prevent sample defrosting) were ground to a fine powder in liquid nitrogen and each sample was extracted with 200μl methanol:isopropanol:acetonitrile (50:49:1, v:v:v) using vortexing and ultrasonication during 30min. Sample extracts were centrifuged at 13,000rpm for 10min at 4°C, the supernatant was collected, and the pellet re-extracted with 200μl of the same solvent. Supernatants were pooled and filtered with 0.22μm PTFE filters (Phenomenex, Torrance, United States), then transferred to HPLC vials and injected into the UHPLC-MS/MS. Phytohormones, including ABA, jasmonic acid (JA), salicylic acid (SA), the auxin indole-3-acetic acid (IAA), the gibberellins GA_1_, GA_3_, GA_4_, and GA_7_, the cytokinins *trans*-zeatin (Z), its riboside *trans*-zeatin riboside (ZR), 2-isopentenyl adenine (2iP) and its riboside isopentenyl adenosine (IPA), and the ethylene precursor 1-amynocyclopropane-1-carboxylic acid (ACC), were separated using an elution gradient on a reverse-phase UHPLC system and quantified using tandem mass spectrometry in multiple reaction monitoring mode, as described by Müller and Munné-Bosch (2011). Recovery rates were calculated for each hormone on every sample by using the deuterium-labeled compounds as internal standards, including d_6_-2iP, d_6_-IPA, d_5_-Z, d_5_-ZR, d_5_-IAA, d_2_-GA_3_, d_2_-GA_4_, d_4_-ACC, d_6_-ABA, d_4_-SA, and d_5_-JA, which were purchased from OlChemim Ltd. (Olomouc, Czech Republic).

### Statistical Analysis

For general fruit quality characteristics, 10 replicates were analyzed for each treatment and time point. As for hormonal profiling, six replicates were used for each treatment and time point. Data were analyzed by two-way ANOVA using the SPSS 25.0 statistical package. Multiple comparison tests were carried out with Tukey’s HSD *post-hoc* test. In all cases, differences were considered significant at a probability level of *p*<0.05.

## Results

### Effect of Water Supply on Leaf Physiology in Vines

**Figure 1:**
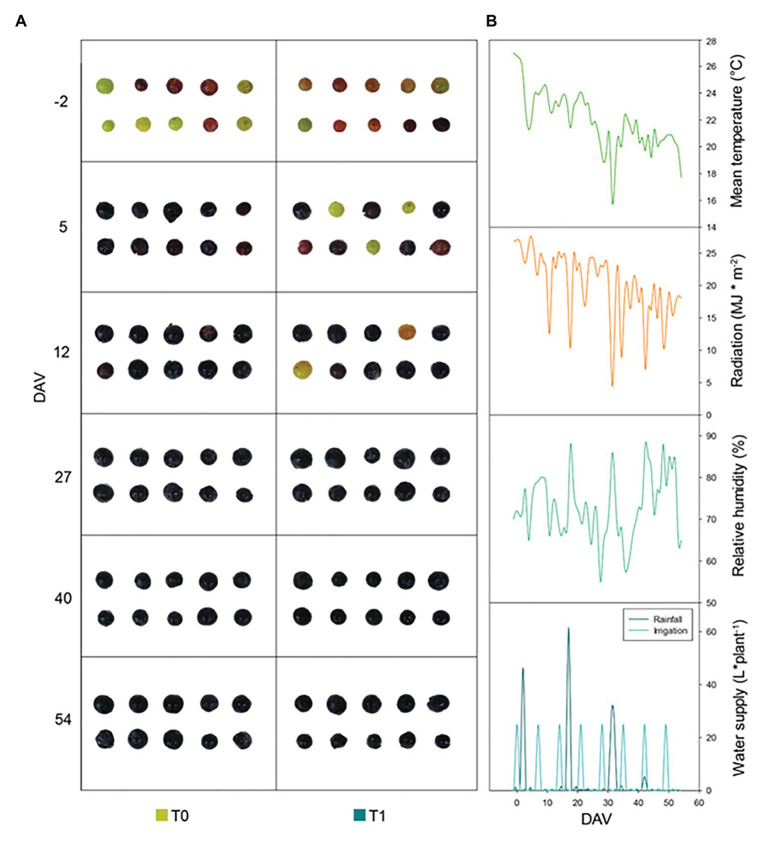
Phenotypic and climatic variations during the study. **(A)** Representative images of “Merlot” grapes phenotypic evolution throughout ripening for non-irrigated (T0) and irrigated (T1) vineyards. **(B)** Climatic conditions in the experimental site during the season, including daily mean temperature, solar radiation, relative humidity, and water supply. Note that T0 vines received rainwater only, while T1 vines received rainwater plus 25Lweek^−1^ vine^−1^. DAV, days after *veraison*.

The differential water supply treatment that was established between T0 and T1 vines, with T1 vines receiving an additional water supply of 25Lweek^−1^ vine^−1^ (Figure 1) between *veraison* (August 10th, 2019) and harvest (October 3rd, 2019) led to small changes in leaf water status (Supplementary Figure S1). The RWC decreased progressively over the season in both treatments, reaching minimum values of 75% at 40 DAV in T0 vines and 76% at harvest in T1 vines (Supplementary Figure S1). Water irrigation improved leaf water contents (as indicated by both RWC and H) at 40 DAV in T1 vines compared to T0 vines, while it did not influence proline contents, LMA, and Fv/Fm ratios (Supplementary Figure S1). Despite mean daily temperatures and maximum solar radiation decreased over the season from August to October (Figure 1), the Fv/Fm ratio, which is indicative of photoinhibitory damage to PSII, was progressively reduced from *ca.* 0.75 at −2 DAV to *ca.* 0.70 at harvest both in T0 and T1 vines (Supplementary Figure S1).

### Assessment of Hormones in Whole Fruits During Grape Berry Ripening

**Figure 2:**
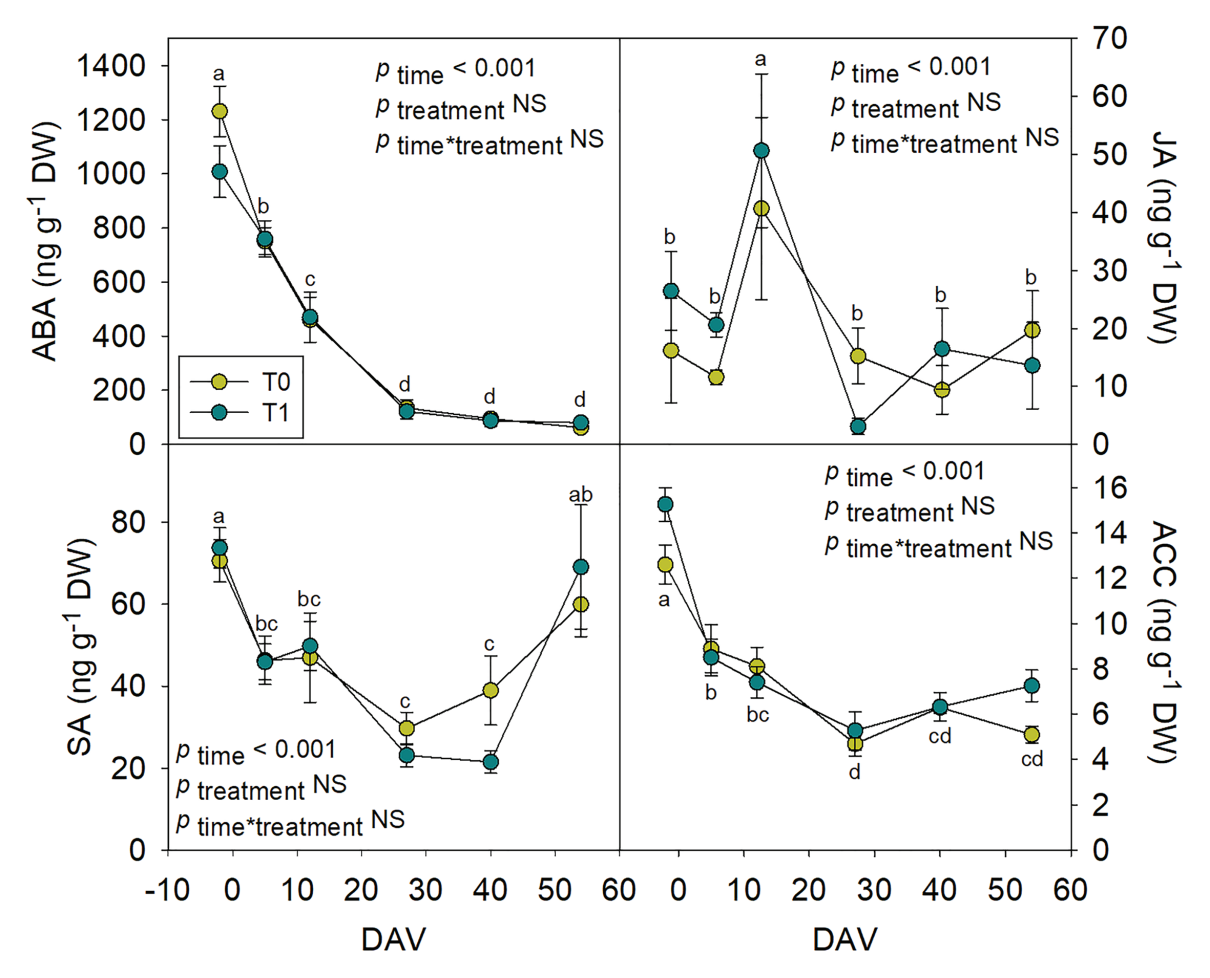
Endogenous variations in abscisic acid (ABA), jasmonic acid (JA), salicylic acid (SA), and the ethylene precursor 1-aminocyclopropane-1-carboxylic acid (ACC) in whole berries of “Merlot” grapes during ripening in non-irrigated (T0) and irrigated (T1) vineyards. ABA, JA, SA, and ACC contents in grapes throughout ripening are shown. Vertical bars indicate SE of six replicates. Different letters denote significant differences among sampling dates (Tukey’s HSD *post-hoc* test *p*<0.05). No significant differences between treatments were observed (*p*>0.05).

**Figure 3:**
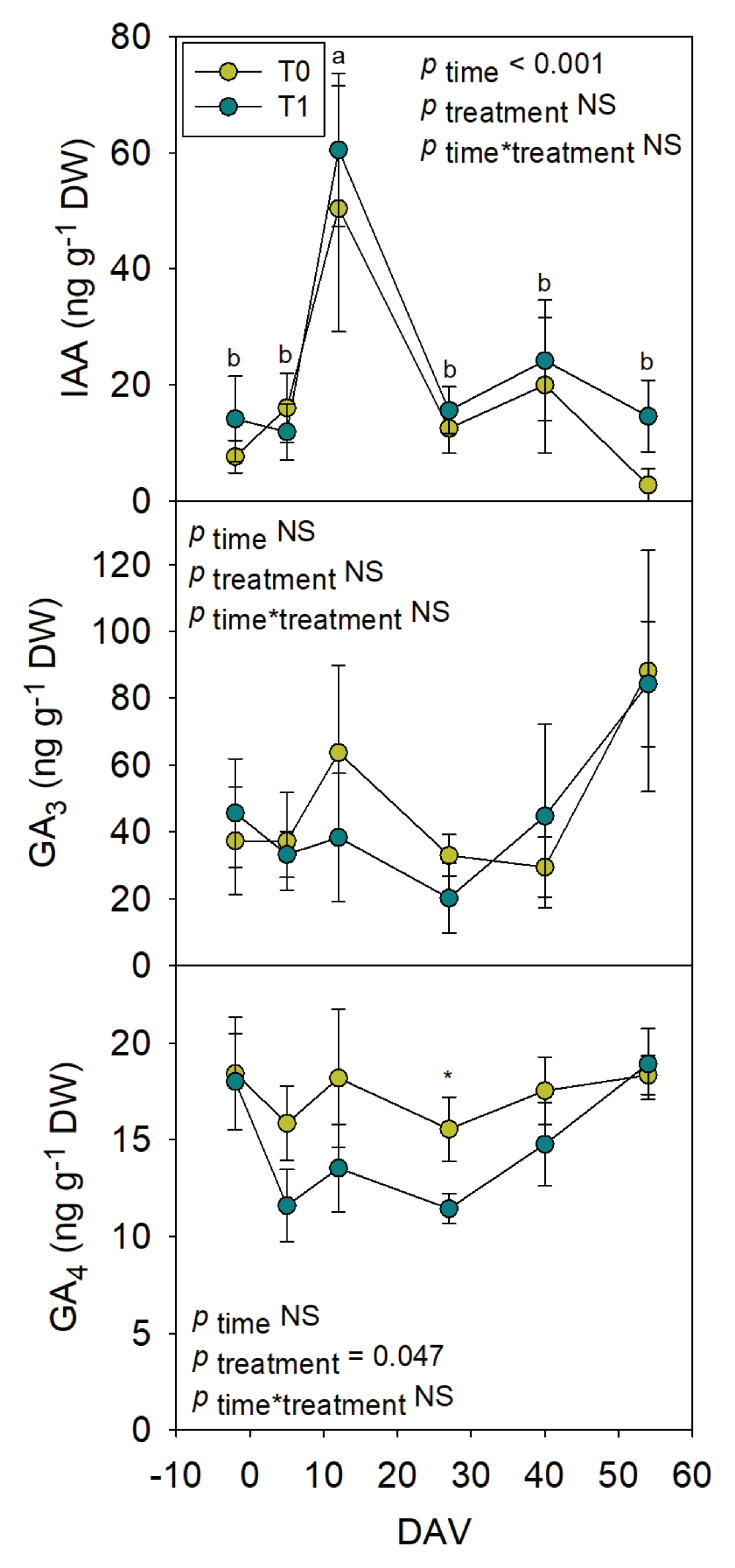
Endogenous variations in auxin and gibberellins in whole berries of “Merlot” grapes during ripening in non-irrigated (T0) and irrigated (T1) vineyards. Indole-3-acetic acid (IAA), GA_3_, and GA_4_ contents in grapes throughout ripening are shown. Vertical bars indicate SE of six replicates. Different letters denote significant differences among sampling dates (Tukey’s HSD *post-hoc* test *p*<0.05). The asterisks indicate significant differences between treatments (*p*<0.05).

**Figure 4:**
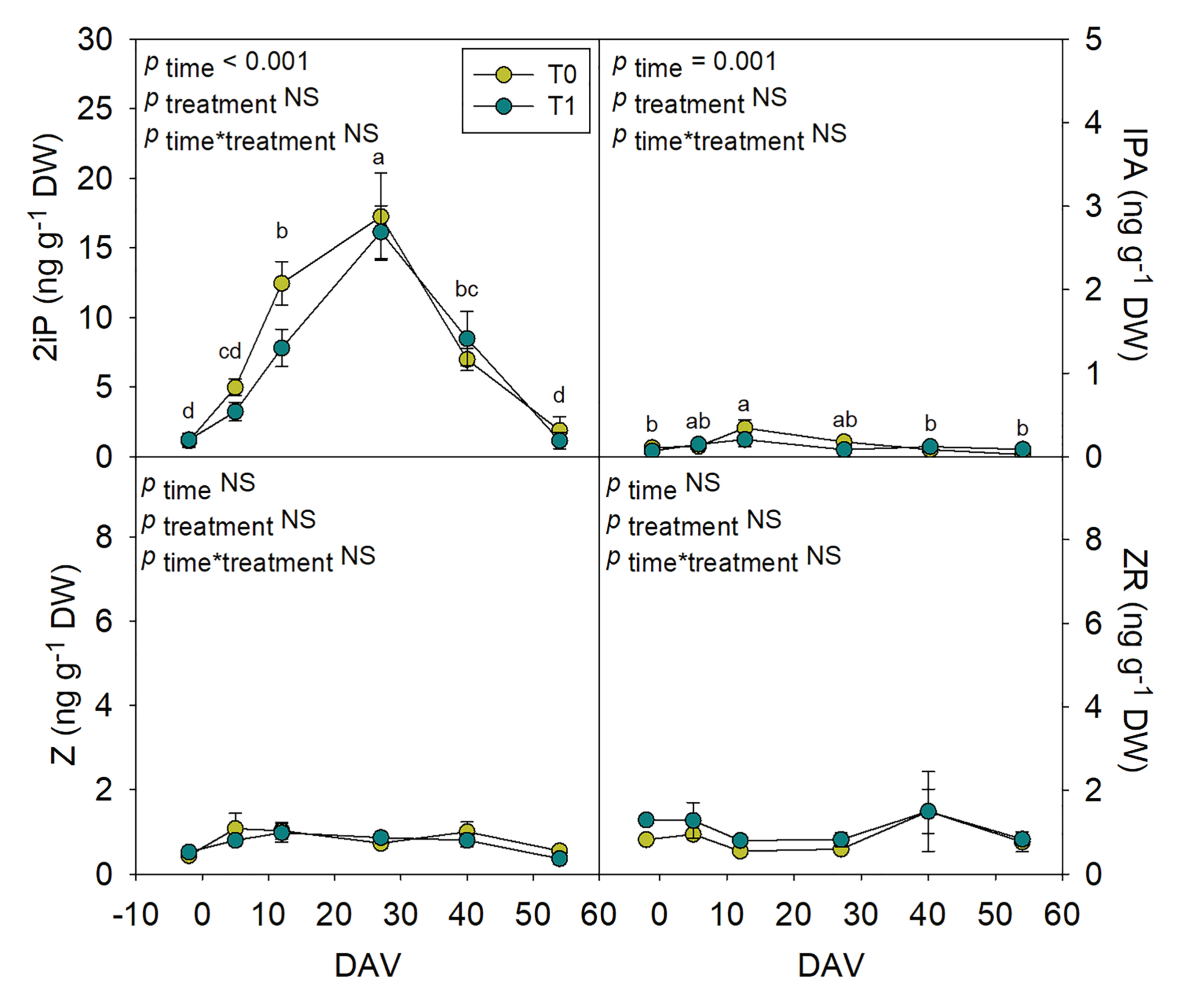
Endogenous variations in cytokinins in whole berries of “Merlot” grapes during ripening in non-irrigated (T0) and irrigated (T1) vineyards. 2-isopentenyl adenine (2iP), isopentenyl adenosine (IPA), *trans*-zeatin (Z), and *trans*-zeatin riboside (ZR) contents in grapes throughout ripening are shown. Vertical bars indicate SE of six replicates. Different letters denote significant differences among sampling dates (Tukey’s HSD *post-hoc* test *p*<0.05). No significant differences between treatments were observed (*p*>0.05).

Hormones related to berry growth and ripening showed different patterns during the season, without significant differences between water treatments, except for GAs. While ABA and ACC contents peaked at −2 DAV and decreased progressively until harvest, JA peaked at 12 DAV and SA peaked at both −2 DAV and 54 DAV (Figure 2). The auxin IAA followed a similar pattern to that described for JA, the contents of both hormones being 5-fold higher at 12 DAV than at −2 DAV (Figure 3). As for GAs, although endogenous contents of GA_3_ were 5-fold higher than those of GA_4_ in grapes berries, the latter were significantly smaller in T1 compared to T0 vines, thus GA_4_ contents being reduced in whole berries of irrigated vines (Figure 3). This difference between treatments was statistically significant (*p*=0.047), the *post-hoc* test showing a significant difference at 27 DAV (*p*<0.05, Figure 3). GA_1_ and GA_7_ were found below the quantification limit. All cytokinins showed a similar response over the season being present at low amounts, except 2iP, which was present at comparatively much higher concentrations, with a progressive increase until 27 DAV, when the contents were 14-fold higher than those observed at −2 DAV, and then a sharp decrease occurred until harvest (Figure 4).

### Effect of Water Supply on Grape Growth Dynamics

**Figure 5:**
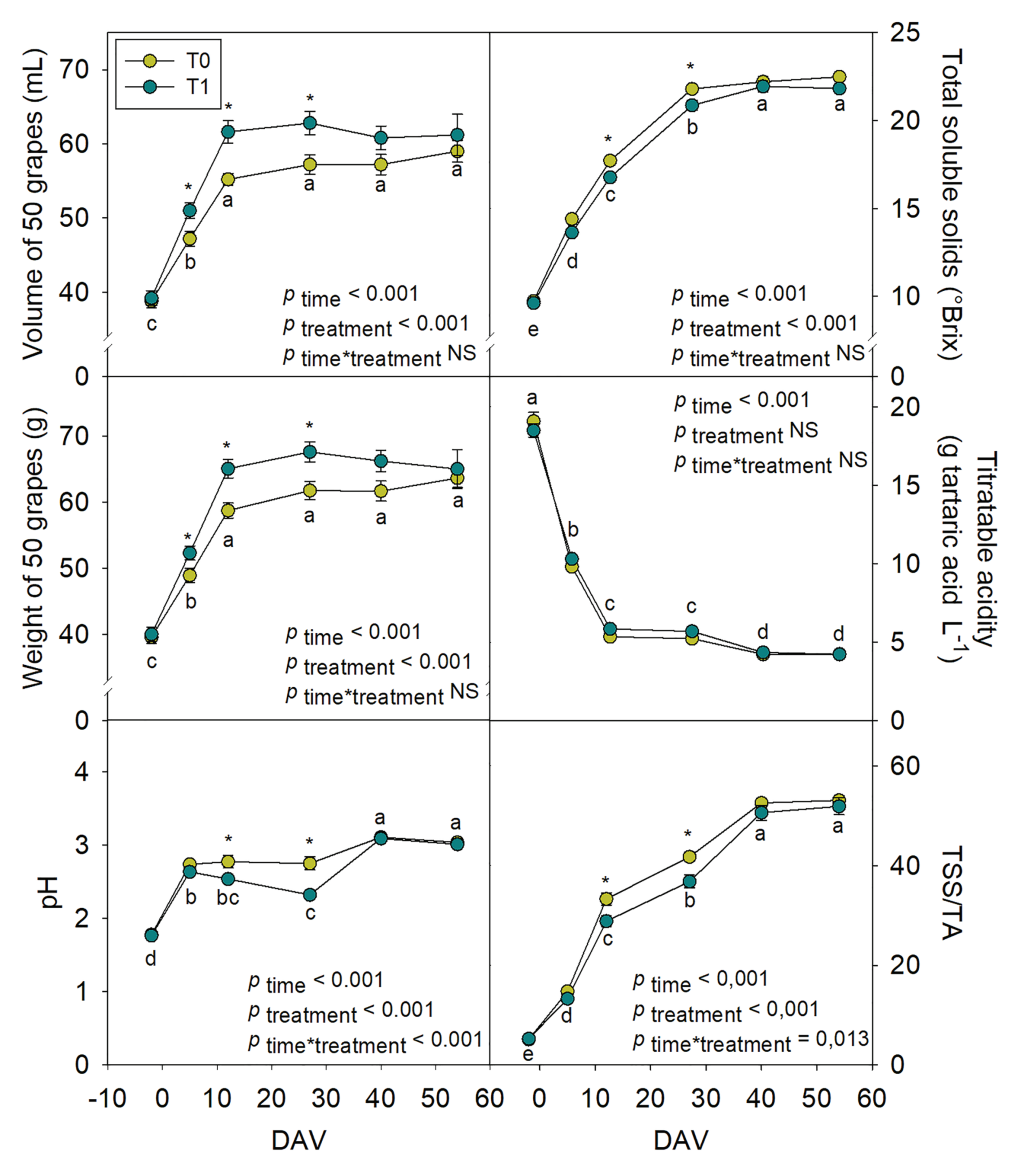
Variations in “Merlot” berry quality characteristics during ripening in non-irrigated (T0) and irrigated (T1) vineyards. Quality parameters measured included grape volume (ml), weight (g fresh matter), pH, total soluble solids (TSS, °Brix), titratable acidity (TA, g L^−1^ tartaric acid) and the TSS/TA ratio. Vertical bars indicate SE of 10 replicates, and 50 berries were used from each replicate. Different letters denote significant differences among sampling dates (Tukey’s HSD *post-hoc* test *p*<0.05). The asterisks indicate significant differences between treatments (*p*<0.05).

The differential water supply treatment that was established to evaluate if modifications in water regimes could affect growth dynamics and berry composition through alterations in hormonal composition revealed important differences in the biochemistry and physiology of fruits. Growth dynamics showed transient but significant differences between treatments, irrigation treatment promoting the fruit biomass increase of grapes over the season (Figure 5). Grapes weight and volume increased progressively over the season, being up to 12 and 11% higher, respectively, from 5 to 27 DAV in irrigated compared to non-irrigated vines. Nevertheless, at harvest time (54 DAV), no significant differences were found in grapes treated with different water regimes. At the same time, pH and TSS also increased during ripening, with 16% lower values of both parameters at 12 and 27 DAV in T1 compared to T0 vines. While TA decreased progressively without significant differences among treatments, TSS and the ratio TSS/TA steadily increased, with higher values for grapes at 12 and 27 DAV in T0 compared to T1 vines. However, no significant differences were found for grapes treated with different water treatments at harvest (Figure 5). The effects of the differential water regimes on berry growth dynamics and composition were accompanied by changes in leaf water contents. Although differences were very small, both RWC and H were slightly improved at 40 DAV in T1 compared to T0 vines. This improvement was, however, small and transient and other leaf stress markers, such as proline contents, LMA and Fv/Fm were not affected by water supply treatments (Supplementary Figure S1).

### Differentiated Hormonal Content in Leaves and Berry Tissues

**Figure 6:**
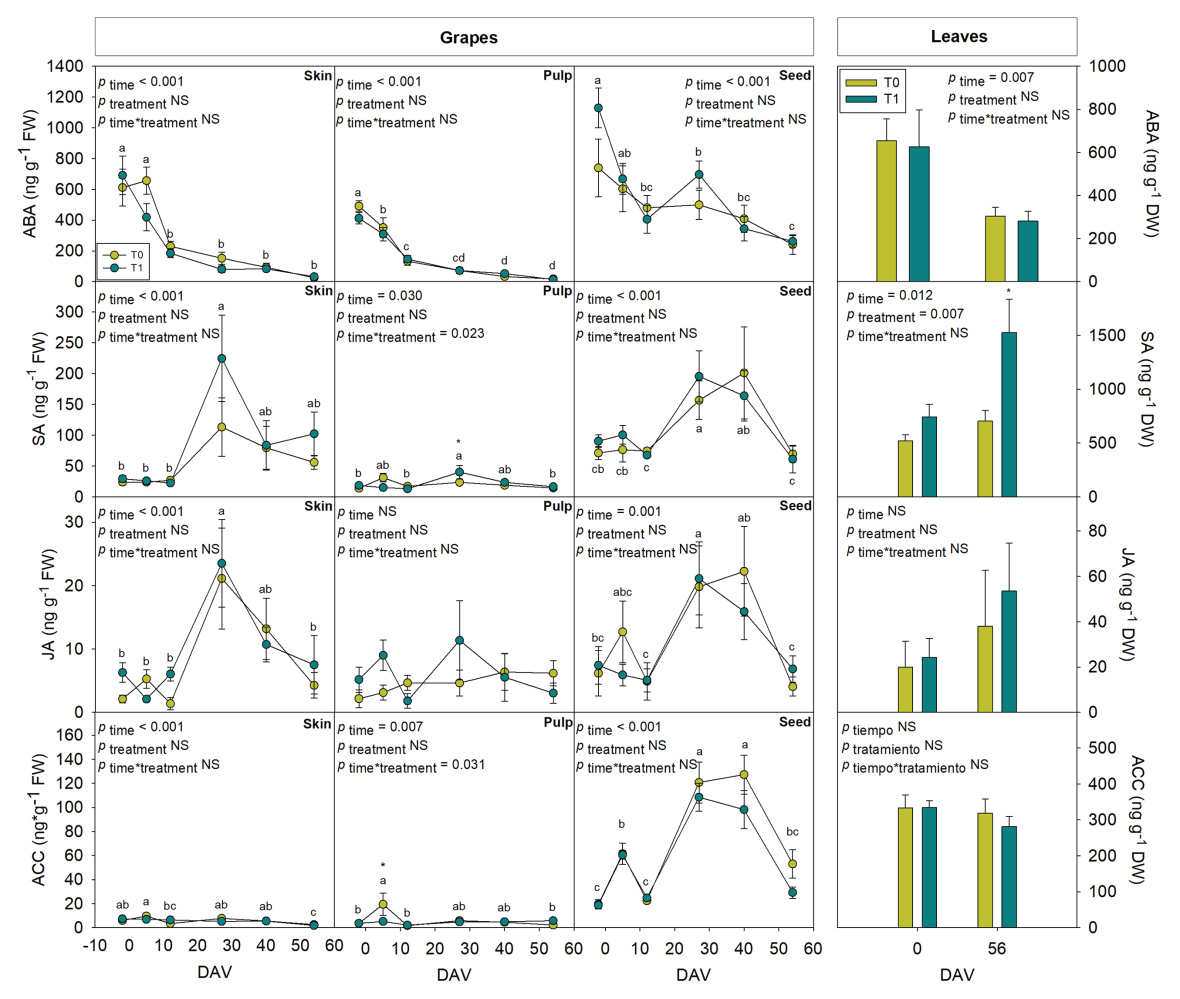
Tissue-specific endogenous variations in ABA, JA, SA, and the ethylene precursor, 1-aminocyclopropane-1-carboxylic acid (ACC) in berries of “Merlot” grapes during ripening in non-irrigated (T0) and irrigated (T1) vineyards. ABA, SA, JA, and ACC contents in grape tissues, including the skin, pulp, and seeds throughout ripening are shown. Hormonal contents in leaves at the beginning and the end of the season are shown for comparison. Vertical bars indicate SE of six replicates. Different letters denote significant differences among sampling dates (Tukey’s HSD *post-hoc* test *p*<0.05). The asterisks indicate significant differences between treatments (*p*<0.05).

**Figure 7:**
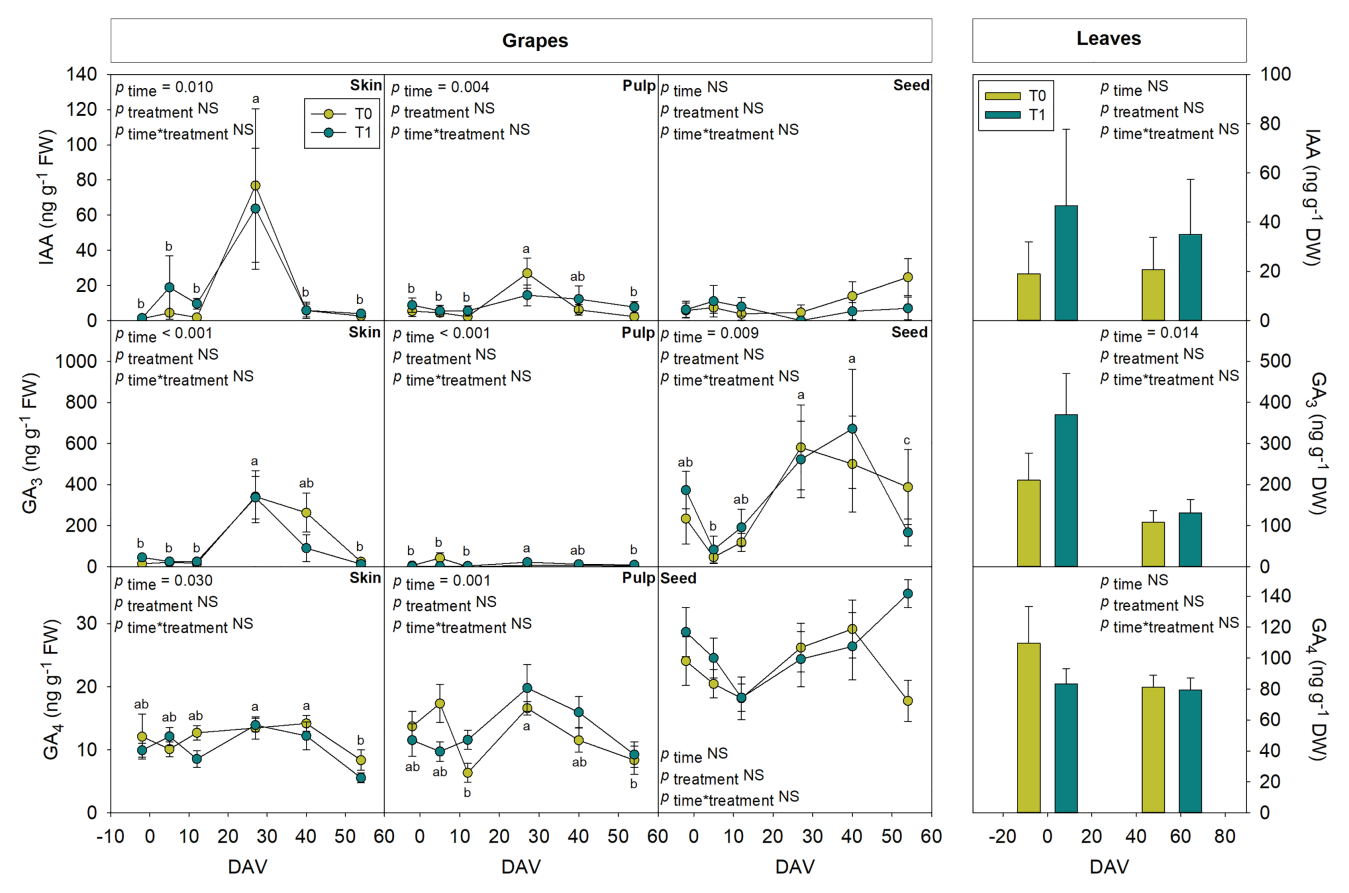
Tissue-specific endogenous variations in auxin and gibberellins in berries of “Merlot” grapes during ripening in non-irrigated (T0) and irrigated (T1) vineyards. Indole-3-acetic acid (IAA), GA3, and GA4 contents in grape tissues, including the skin, pulp and seeds throughout ripening are shown. Hormonal contents in leaves at the beginning and the end of the season are shown for comparison. Vertical bars indicate SE of six replicates. Different letters denote significant differences among sampling dates (Tukey’s HSD *post-hoc* test *p*<0.05). The asterisks indicate statistically significant differences between treatments (*p*<0.05).

**Figure 8:**
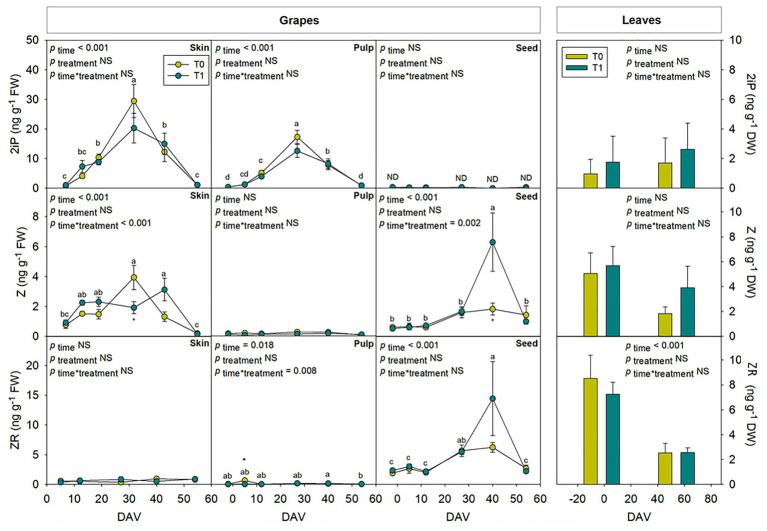
Tissue-specific endogenous variations in cytokinins in berries of “Merlot” grapes during ripening in non-irrigated (T0) and irrigated (T1) vineyards. 2-isopentenyl adenine (2iP), isopentenyl adenosine (IPA), *trans*-zeatin (Z) and *trans*-zeatin riboside (ZR) contents in grape tissues, including the skin, pulp and seeds throughout ripening. Hormonal contents in leaves at the beginning and the end of the season are shown for comparison. Vertical bars indicate SE of six replicates. Different letters denote significant differences among sampling dates (Tukey’s HSD *post-hoc* test *p*<0.05). The asterisks indicate statistically significant differences between treatments (*p*<0.05).

Tissular hormonal analysis was carried out to determine the contribution from skin, pulp, and seeds to the hormonal contents of whole berries throughout ripening, establishing a comparison as well with hormonal profile from leaves at the beginning and the end of the experiment. Results showed a differential behavior between tissues regarding both hormonal contents and dynamics throughout the season. In leaves, only SA showed an increase with additional irrigation at the end of the season, with 2.4-fold higher contents in T1 compared to T0 vines (Figure 6). On the other hand, even though ABA (Figure 6), GA_3_ (Figure 7), and Z (Figure 8) decreased their contents at the end of the season, no significant differences were found in hormonal contents of leaves between different water treatments. Furthermore, differences in GA_4_ contents in whole fruits due to the differential water regime (Figure 3) could not be attributed to any tissue (Figure 7).

Seeds showed a high activity in hormonal accumulation, with high contents of the ripening promoting phytohormones ABA and ACC throughout the season in comparison what those observed in skin and pulp (Figure 6). Likewise, phytohormones related to cell growth and expansion, including gibberellins GA_3_ and GA_4_ (Figure 7), together with Z and its precursor ZR (Figure 8), also accumulated at higher amounts in seeds. In contrast, the skin contained more IAA and 2iP than the pulp and seeds, with endogenous variations showing a similar pattern to that observed in the pulp (Figures 7, 8). Nevertheless, in general, the pulp accumulated lower contents of all phytohormones, with very low amounts of ACC, IAA, GA_3_, Z, and ZR.

Phytohormones showed similar dynamics at the tissular level throughout ripening between both water regimes, with some exceptions. JA and SA showed a similar pattern in berry skin, with a sharp increase at 27 DAV (Figure 6). Likewise, JA, SA, and ACC also differentially accumulated in grape seeds from 27 DAV onward, with an acute decrease at harvest (Figure 6). The evolution of growth-promoting phytohormones such as IAA, Z, and 2iP in the skin showed a similar pattern between them with increases around 27 DAV, but also with some differences between treatments, with a slight delay in Z accumulation in grapes skin in T1 compared to T0 vines. Indeed, the contents of Z, an active cytokinin, increased earlier (between 5 and 12 DAV) to later keep more constant in the skin from T1 compared to T0 vines. Moreover, transient increases in Z and ZR were found at 40 DAV in grape seeds, particularly in irrigated vines, where the accumulation of Z was 3-fold higher in T1 compared to T0 vines (Figure 8).

## Discussion

Ripening of grape berries is a complex process whose beginning occurs at *veraison* and it is characterized by the onset of sugar accumulation, a rapid pigmentation of berries due to anthocyanin synthesis and accumulation in red varieties, a decrease in organic acids content, and a subsequent increase of pH and berry softening (Conde et al., 2007). Changes occurring during this stage directly affect grapes and wine quality, since fundamental chemical characteristics, such as sugar content, TSS/TA ratio, titratable acidity, and accumulation of phenolic compounds are strongly determined by the evolution of ripening (Conde et al., 2007; Fortes et al., 2015). Water deficit is one of the most widespread practices worldwide to increase grapes quality, especially when applied at the onset of ripening, and it has been widely studied regarding its effects on grapes composition (Santos et al., 2005; Intrigliolo and Castel, 2009; Cáceres-Mella et al., 2018; Talaverano et al., 2018). It has been observed that a severe water stress can originate a sharp decrease in both yield and grape quality, while a mild water stress can help to improve quality, but usually decreasing yield (Poni et al., 2018). Grapevines are well adapted to Mediterranean conditions with a high drought tolerance but in a climate change context, it is expected that growth and ripening will be affected by water scarcity. In order to cope with potential drought damage, slight irrigation treatments are now arising as vineyard management strategies to cope with drought stress. In the case of this study, T1 treatment induced a transient delay in grape berry ripening that was observed in grape berry quality with a decrease in TSS, pH, and TSS/TA from 12 to 27 DAV. Likewise, there was an increase in grape weight and volume from 5 to 27 DAV, without significant differences between treatments at harvest. Similar effects were also observed by Zarrouk et al. (2012), where regulated deficit irrigation did not show any difference regarding fruit fresh weight, TSS, TA, or TSS/TA ratio at harvest. Buesa et al. (2017) also described a delay in fruit ripening when vines were fully irrigated in comparison to the water-stressed vines, but only during the driest seasons. This suggests that a slight irrigation addition during dry and warm seasons could help delay the technologic maturity and avoid its decoupling with phenolic maturity (Palliotti et al., 2014). In our study, T0 vines were exposed to a mild stress resulting from the combination of soil water deficit, high temperatures, and high solar radiation, the first increasing over the season, while the two latter decreasing from *veraison* (August 10th, 2019) to harvest (October 3rd, 2019). Water irrigation in T1 vines only improved leaf water contents at 27 DAV but accelerated grape berry growth at 5, 12, and 27 DAV, while the Fv/Fm ratio was not improved, thus suggesting T1 vines were exposed to a more moderate soil water deficit than T0 vines, despite high vapor pressure deficits and solar radiation at midday still infringed stress. Indeed, ABA contents above 200ng/g DW in leaves of both T0 and T1 vines at harvest still indicate some degree of water stress in both treatments (Gambetta et al., 2020). Since the ripening process is highly regulated by phytohormones, whose contents vary differentially within the different developmental stages of grapes, we examined whether growth- and ripening-related phytohormones could vary in grape berries within different water regimes.

Abscisic acid contents showed a decrease throughout the season in all grape tissues, including skin, pulp, and seeds, which agrees with Fortes et al. (2015), who observed decreases in ABA contents at mid ripe stages and harvest. It has been widely described an increase in ABA levels around *veraison*, coinciding with the increase in sugar and anthocyanins accumulation and berries softening, which suggest that ABA plays a key role at the onset of ripening (Davies and Böttcher, 2009; Kuhn et al., 2013; Koyama et al., 2018). Ethylene precursor ACC has also been proposed as a ripening promoter, since an increase of ACC oxidase, an enzyme involved in ethylene production (Agudelo-Romero et al., 2013) has a peak of accumulation just before *veraison* (Deluc et al., 2007; Pilati et al., 2007) and some isoforms of this enzyme are thought to be active after *veraison* (Agudelo-Romero et al., 2013). Our results showed a decrease in ACC content after *veraison*, with huge different concentrations of this ethylene precursor between grapes tissues, seeds displaying the highest contents in late ripe stages, both at 27 and 40 DAV.

Indole-3-acetic acid is thought to be an inhibitor of grape berry ripening but necessary for the onset of the process (Böttcher et al., 2010) and its content remains low after *veraison* (Zhang et al., 2003). In the present study, there was a clear peak of IAA at 27 DAV in whole grapes, grapes skin, and pulp, highlighting a major contribution of skin in the auxin fruit contents. Regarding cytokinins, it has been observed that their concentration is steadily low during grape ripening (Zhang et al., 2003; Davies and Böttcher, 2014), coinciding with observations in the present study, except for 2iP, which showed a progressive increase until 27 DAV and a subsequent decrease, with similar patterns in full berries, skin, and pulp. Interestingly, while the skin accumulated more active cytokinins (2iP and Z), the seeds accumulated more zeatin-type cytokinins (Z and ZR). As both 2iP and ZR can serve as precursors for Z, but only 2iP seems to have some cytokinin activity (together with Z), is it necessary to perform further studies to better understand the spatiotemporal dynamics of cytokinins, their interconversion, and their activity in grape berries. Furthermore, the most striking differences caused by the differential water regime were observed for Z, which increased earlier (between 5 and 12 DAV) but later, kept more constant in the skin from T1 compared to T0 vines. Z contents were also higher at 40 DAV in seeds of T1 compared to T0 vines. It is, therefore, tempting to speculate that the differential grape berry growth dynamics observed between vineyards exposed to a differential water treatment might be associated with skin and seed cytokinin contents. Although this might be true for the skin, which showed a differential behavior between T1 and T0 vines early in fruit development, paralleling changes in growth dynamics between water regime treatments, growth-related differences in the fruit did not coincide with differences in cytokinin contents in the seeds at 40 DAV. It is very likely that changes in skin cytokinin contents might be associated with the transient differences in growth and ripening due to the differential water regimes (favoring accelerated cell division and increasing the sink strength of seeds in irrigated vines), while cytokinin changes later in development in the seeds might be more associated with processes of seed maturation (Jameson and Song, 2015). It is noteworthy that early changes in fruit growth dynamics were not accompanied by variations in the leaf water contents, while small but significant increases in leaf water contents occurred at 40 DAV. Further research is, therefore, needed to better understand how cytokinin contents govern source-sink relationships between leaves and fruits, and how cytokinin metabolism is regulated in different fruit tissues, at different stages of grape berry ripening in vineyards exposed to various water regime treatments.

The efforts of studying the biological significance of GAs in grapes have been focused in table grapes and mainly in seedless varieties, since they promote cell division and expansion (Zhang et al., 2003), and they have been proposed as putative negative regulators for berries ripening (Davies and Böttcher, 2009). However, there is still a lack of clear causal evidence to reveal its role in grape berry growth and ripening. In this study, seeds also showed higher GA_4_ contents than skin and pulp, suggesting an active role of this tissue in grapes growth dynamics, possibly interacting with cytokinins, although further research is needed to clarify this point. Although GA_4_ contents slightly, but significantly differed in whole fruits from T1 and T0 vines, no differences in the endogenous contents of this phytohormone were observed in the fruit tissues examined between water regime treatments. The hydration of fruits was not altered by a differential water regime treatment in T1 and T0 vines (Supplementary Figure S2), but the pH and TSS did. Furthermore, the highest reductions in GA_4_ contents coincided with the lowest reduction in pH of grape berries of T1 compared to T0 vines. Indeed, a positive correlation was found between GA_4_ contents and pH during development and ripening of sweet cherries, another non-climacteric fruit (Teribia et al., 2016), in which study, the pH correlated positively with IAA, cytokinins and GAs, and negatively with ABA.

In our study, endogenous hormonal contents did not differ in the pulp of grape berries between T1 and T0 vines, except for SA contents, which were higher in the pulp of fruits of T1 compared to those of T0 vines at 27 DAV. It is still unknown if endogenous changes in SA in the pulp might affect grape berry quality characteristics. It is also unknown how the additional water received by T1 vines might affect the soil on which vines were grown, which is known to impart a unique quality to the grapes and wine, but higher SA contents were also observed in leaves of T1 vines compared to T0 vines at the end of the study, thus suggesting that T1 vines might be more exposed to biotic stress factors than T0 vines because of the additional water regime received. How the soil microbiome may be affected by water irrigation in vineyards, and how this might influence the activation of biotic and abiotic stress tolerance mechanisms in leaves and grape berries requires further investigation, although it is already known that the soil microbiome affects the grapevine-associated microbiome (Zarraonaindia et al., 2015).

## Conclusion

The dynamics of fruit development and ripening of “Merlot” grape, as well as their underlying hormonal control may be affected by slight changes in water supply. Our study underlines the importance of spatiotemporal approaches for a better understanding of fruit ripening, since the hormonal control of developmental processes (not only fruit ripening, but also changes in growth dynamics caused by differential water regimes) appear to be strongly tissue specific. A challenge of current viticulture is better understanding how differential water regimes influence grape berry ripening and wine quality. Considering the contribution of various tissues to the hormonal modulation of grape development and ripening inferred from the present study, it is of particular interest to further explore the specific role not only of the skin but also of the pulp and seeds in fruit ripening and quality characteristics. Also, of interest will be to further explore how fruit ripening and quality may be influenced by processes occurring not only locally (in the grape berry tissues), but also systemically, either in leaves (with a special emphasis on source-sink relations between leaves and fruits) or in the rhizosphere (plant-microbiome interactions).

## Data Availability Statement

The raw data supporting the conclusions of this article will be made available by the authors, without undue reservation.

## Author Contributions

CR-P and SM-B conceived and designed the experiments. CR-P conduced the physiological and biochemical analyses with the guidance of PM and SM-B. CR-P wrote the manuscript with the help of PM and SM-B. CR-P prepared all the figures. All authors contributed to the discussion of ideas, revised, and approved the final manuscript.

### Conflict of Interest

The authors declare that the research was conducted in the absence of any commercial or financial relationships that could be construed as a potential conflict of interest.
