# Supplementary material for: Tissue-Specific Hormonal Variations in Grapes of Irrigated and Non-irrigated Grapevines (Vitis vinifera cv. “Merlot”) Growing Under Mediterranean Field Conditions
Source: Front Plant Sci. 2021 Feb 1;12:621587. doi: 10.3389/fpls.2021.621587 (PMC7882616; doi:10.3389/fpls.2021.621587)
Supplement: Supplementary file 1 [file Presentation_1.pptx]

## Slide 1
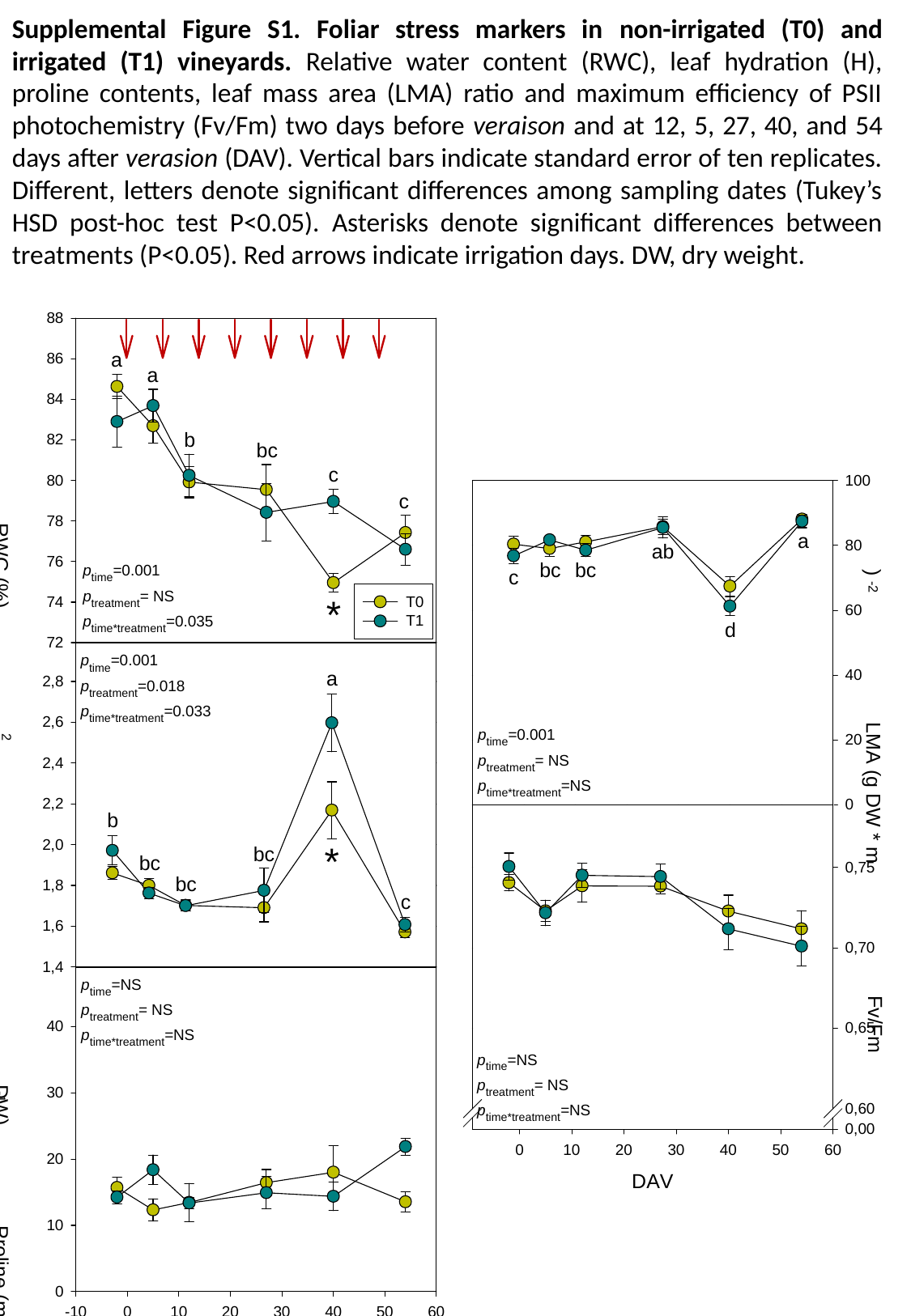

Supplemental Figure S1. Foliar stress markers in non-irrigated (T0) and irrigated (T1) vineyards. Relative water content (RWC), leaf hydration (H), proline contents, leaf mass area (LMA) ratio and maximum efficiency of PSII photochemistry (Fv/Fm) two days before veraison and at 12, 5, 27, 40, and 54 days after verasion (DAV). Vertical bars indicate standard error of ten replicates. Different, letters denote significant differences among sampling dates (Tukey’s HSD post-hoc test P<0.05). Asterisks denote significant differences between treatments (P<0.05). Red arrows indicate irrigation days. DW, dry weight.

## Slide 2
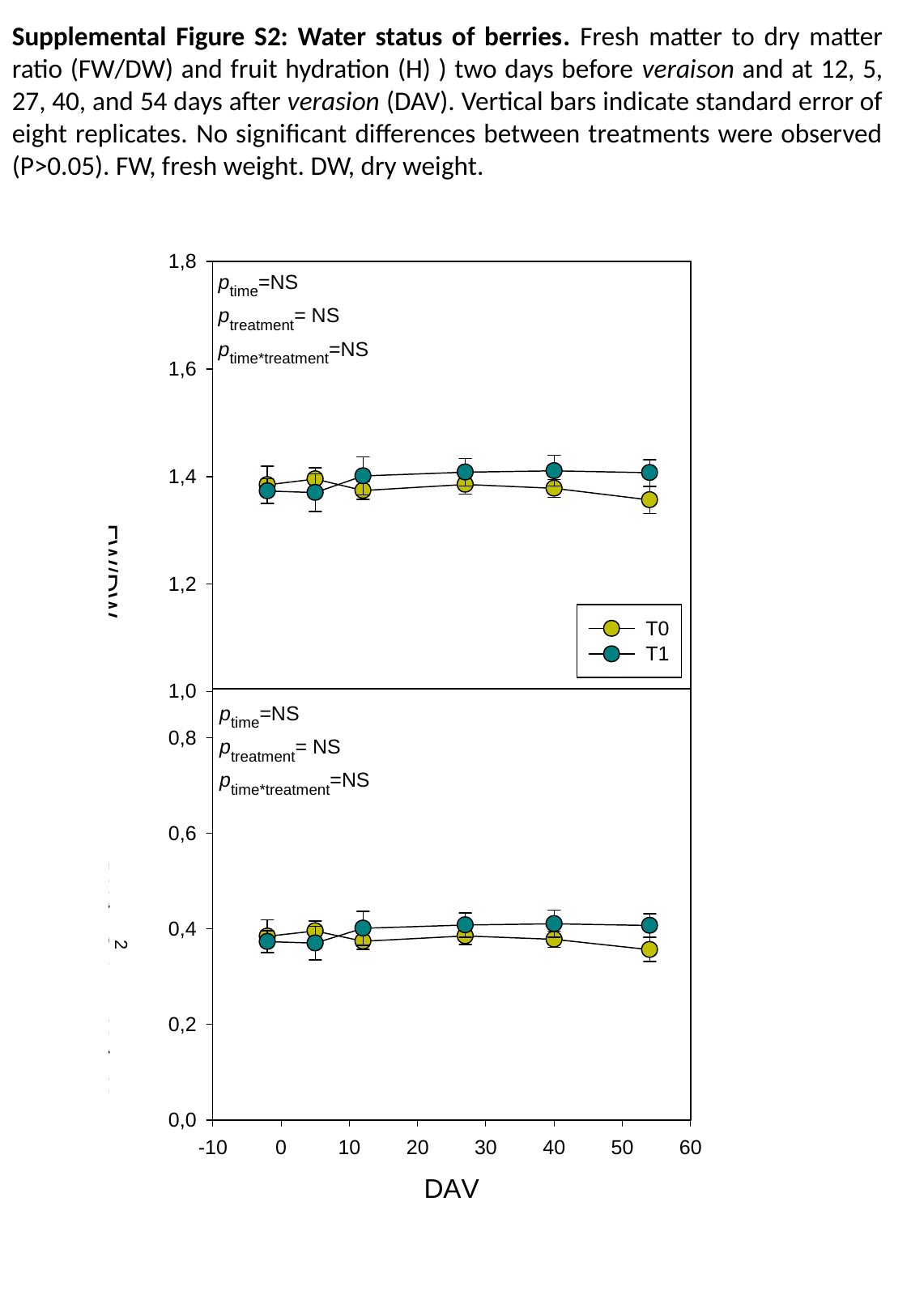

Supplemental Figure S2: Water status of berries. Fresh matter to dry matter ratio (FW/DW) and fruit hydration (H) ) two days before veraison and at 12, 5, 27, 40, and 54 days after verasion (DAV). Vertical bars indicate standard error of eight replicates. No significant differences between treatments were observed (P>0.05). FW, fresh weight. DW, dry weight.
